# Mobile phone access and preferences among medical inpatients at an urban Canadian hospital for post-discharge planning: A pre-COVID-19 cross-sectional survey

**DOI:** 10.3389/fdgth.2022.928602

**Published:** 2022-11-11

**Authors:** Maryam AboMoslim, Abdulaa Babili, Niloufar Ghaseminejad-Tafreshi, Matthew Manson, Fanan Fattah, Samia El Joueidi, John A. Staples, Penny Tam, Richard T. Lester

**Affiliations:** ^1^ Division of Infectious Disease, Faculty of Medicine, University of British Columbia, Vancouver, BC, Canada; ^2^ Faculty of Public Health and Policy, London School of Hygiene & Tropical Medicine, London, United Kingdom; ^3^ Division of Vancouver Costal Health Research Institutute, Centre for Clinical Epidemiology & Evaluation, Vancouver, BC, Canada; ^4^ Division of General Internal Medicine, Department of Medicine, University of British Columbia, Vancouver, BC, Canada

**Keywords:** mHealth, virtual care, mobile phone penetration, digital health, patient engagement, hospital readmission, health services planning

## Abstract

**Background:**

Digital health interventions are increasingly used for patient care, yet little data is available on the phone access type and usage preferences amongst medical ward inpatients to inform the most appropriate digital interventions post-discharge.

**Methods:**

To identify mobile phone ownership, internet access, and cellular use preferences among medical inpatients, we conducted a researcher-administered survey of patients admitted to five internal medicine units at Vancouver General Hospital (VGH) in January 2020. The survey was administered over 2 days separated by a 2-week period.

**Results:**

A total of 81 inpatients completed the questionnaire. Survey found that 85.2% of survey respondents had mobile phone access where 63.0% owned their own mobile phone, and 22.2% had access to a mobile phone *via* a proxy (or an authorized third-party) such as a family member. All participants with mobile phone access had cellular plans (i.e., phone and text); however, a quarter of respondents did not have data plans with internet access. Survey showed that 71.1% of males owned a mobile phone compared to only 52.8% of females. All participants at a “high” risk of readmission had access to a mobile phone, either as phone-owners or proxy-dependent users.

**Conclusion:**

Access to mobile phones among medical ward inpatients, 85.2%, was comparable to smartphone penetration rates amongst Canadians in 2019, 85.1%. More patients had cellular than data plans (i.e., internet and applications). Understanding patient-specific access is key to informing potential uptake of digital health interventions aimed at using patients' mobile phones (mHealth) from an effectiveness and equity lens.

## Background

The use of mobile phone technology is mushrooming as devices have become more advanced, affordable, and an integral part of life. There are an estimated 4.78 billion unique mobile phone users among the current global population of over seven billion people, giving a penetration rate of approximately 61.4% (1, 2). Furthermore, the digital health market revenue is expected to reach over $536.6 billion by the end of the year 2025 (3). A subsection of this market, Mobile Health (mHealth), is defined by the World Health Organization (WHO) as “mobile and wireless technologies to support the achievement of health objectives” (4). In the course of mHealth adoption as a healthcare delivery tool, mobile phones are increasingly viewed as medical devices by the WHO, U.S., the Food and Drug Administration (FDA), hospital administration, healthcare providers, and insurance companies (5, 6). Moreover, mobile health (mHealth) has proven to be a reliable management and prevention strategy to the COVID-19 pandemic. While digital health technologies existed before the COVID-19 pandemic, COVID-19 significantly accelerated the optimization and adoption of such interventions to target the emerging needs of healthcare delivery during an emergency state with increased pressure on the healthcare facilities, and to deliver care while adhering to public health orders that limited access of the general patient population to acute care facilities, and implementing stay-home orders to maintain social distancing and ensure continuity of care. The pandemic provided the opportunity to explore the use of such interventions within different disease contexts (entire spectrum of healthcare services) (e.g., diabetes and mental health services). The adoption of such digital health interventions for healthcare delivery during COVID-19 was seen in both developed and developing countries, which indicated the universal applicability of such tools within different socioeconomic, political and geographical contexts. This remarkable expansion of the digital health market suggests a move towards mHealth innovations as the new standard of care and highlights the importance of evaluating mobile phone access among patients as they transition in and out of hospitals, community care and other healthcare settings.

Timely access to health information and care is important at the patient, provider, and health system levels given its impact on clinical outcomes, patient satisfaction, and continuity of care (7). As such, innovative healthcare interventions have used mHealth platforms to improve medication adherence, post-discharge transitional care, patient-provider communication, and medical biomarkers such as suppression of viral load among HIV positive individuals and serum glucose in individuals with diabetes (8–12). Despite major advancement in the field of digital health, the uptake of digital interventions has remained below expectations (13, 14). Some worry that the interventions used may not be best tailored to the patients' access and usage, especially among those that are most vulnerable and use a disproportionate amount of health care resources (15). Although many patients have access to mobile phones, little is known about their usage preferences or limitations such as internet access, which may be restricted, particularly for marginalized populations for whom data plans are unaffordable (16). As more mHealth interventions are being deployed in clinical settings (9, 10), it is important to capture and assess patients' usage and preferences to ensure equitable access to health care resources. To this end, patients' mobile phone use patterns and their sentiments towards mHealth interventions remain inadequately researched (9, 10, 17, 18). We sought to learn about mobile phone ownership, internet access, and cellular use preferences among medical ward patients in order to inform the development of a mHealth intervention aimed at improving continuity of care and inform the development of hospital discharge protocols.

## Methods

### Study sample and setting

This study took place on the Internal Medicine Clinical Teaching Units (IM CTU) at Vancouver General Hospital (VGH), a quaternary-care teaching hospital with approximately 700 acute care beds located in the Canadian province of British Columbia (BC). The IM CTU has approximately 114 beds and provides acute medical care to over 4,400 individuals each year. Each unit is assigned Care Management Leads (CMLs) who follow a discharge-planning protocol that consists of the assignment of a Readmission Risk Assessment Score (RRAS) to each patient. The RRAS is based on a hospital prediction model, called the LACE index, and is modified to meet local needs (52). Prior to discharge, patients receive a “Low”, “Moderate”, or “High” RRAS depending on medical risk (such as an exacerbation of a chronic disease) and/or social risk (such as an inability to carry-out self-management activities). Hospital readmissions are not only costly to the healthcare system, but they often place a significant burden on patients and their families (19). As a result, many programs focus on reducing the rate of readmission by implementing targeted interventions for those likely to return to the hospital after discharge (20). Current protocol at VGH is for patients to be referred to a Transitional Services Team (TST) and the Vancouver Community Case Management (VCCM) who follow up with patients post-discharge. Patients with a “moderate” to “high” RRAS receive a follow-up phone call within 48 h of discharge. Post-discharge resources exclude patients with a “low” RRAS, who potentially require support after being discharged from the hospital due to other issues not captured by the RRAS such as low socioeconomic status (11).

### Study inclusion and exclusion criteria

Inclusion criteria consisted of patients who were (1) admitted to one of five IM CTUs; (2) able and willing to provide informed consent; and (3) able to complete the survey in English or French or *via* the aid of a proxy (i.e., present spouse or child). Our target are patients who are discharged from hospital to independent living within the community. Therefore, exclusion criteria consisted of patients who were (1) patients returning to facility-assisted care such as residents of Long-Term Care Facilities (LTCF) or correctional facilities; (2) unable to interact with study staff as determined by the CMLs.

### Data sources

The survey was preloaded on a Wi-Fi-enabled tablet and administered orally by 3 research assistants who were uniformly trained on this survey administration. Members of the research team approached eligible participants in their room, obtained informed consent, and administered the survey, each of which took approximately 5 min to complete. Surveys were administered on January 7th, 2020 and January 23rd, 2020; a 2-week period between survey dates was allotted to decrease chances of surveying the same pool of patients. Participants already surveyed on January 7th, and remaining in the hospital on January 23rd, were excluded the second time to avoid duplicate responses. RRAS as well as patient demographic information, such as gender and year of birth, were accessed *via* the CMLs and de-identified using the assignment of a unique patient identifier. No incentive was provided for participating in this study. The survey form was built on and data was collected using Qualtrics software, Version January 2020.

### Study design and protocol

The study is a cross-sectional observational ten-question orally-administered survey. The questions collected information on patient mobile phone ownership, usage, preferences, and internet access. The goal was to approximate a census by surveying all eligible inpatients on all 5 medical wards. The research team attempted contact with each eligible participant 2 times throughout the day. If a patient is still unavailable (e.g., out of room or asleep), the research team returned the following day for the third and final time.

All survey questions were developed in English, designed by the mHealth Research Group, and audited by a physician, a CML nurse, a leading mHealth researcher and physician, and the Director of Strategic Initiatives at Vancouver Coastal Health (VCH). At least one research assistant present is a fluent French speaker, and prepared to administer the survey in French upon request. To ensure reliable and standardized data collection, research staff received training on the ethical administration of surveys, obtaining informed consent, infection control, etiquette of interacting with marginalized patients, and other study protocols to be upheld throughout the study. This study was approved by the University of British Columbia Behavioral Research Ethics Board (certificate H19-03366).

### Statistical analysis

The analysis in this study was mainly descriptive in design. The purpose of this descriptive analysis is to examine and describe participants' characteristics in relation to phone access and use. Survey data was exported from Qualtrics for analysis. Data cleaning and further data management was carried out in Microsoft Excel (2019). A normality test was conducted to measure the distribution of age. Age variable was reported as median with interquartile range (Median (IQR)). Categorical variables, such as gender, RRAS, and access rates, were reported as frequencies and percentages. A univariate linear regression was used to predict if phone ownership is associated with high, moderate, and low RRAS scores. A Chi-Square test was used to ascertain the results from the linear regression analysis and examine the relationship between phone ownership and RRAS scores. A significance level of 0.05 was predetermined (*a* = 5%), and data was analyzed using R 1.2.5033 (54).

## Results

**Figure 1:**
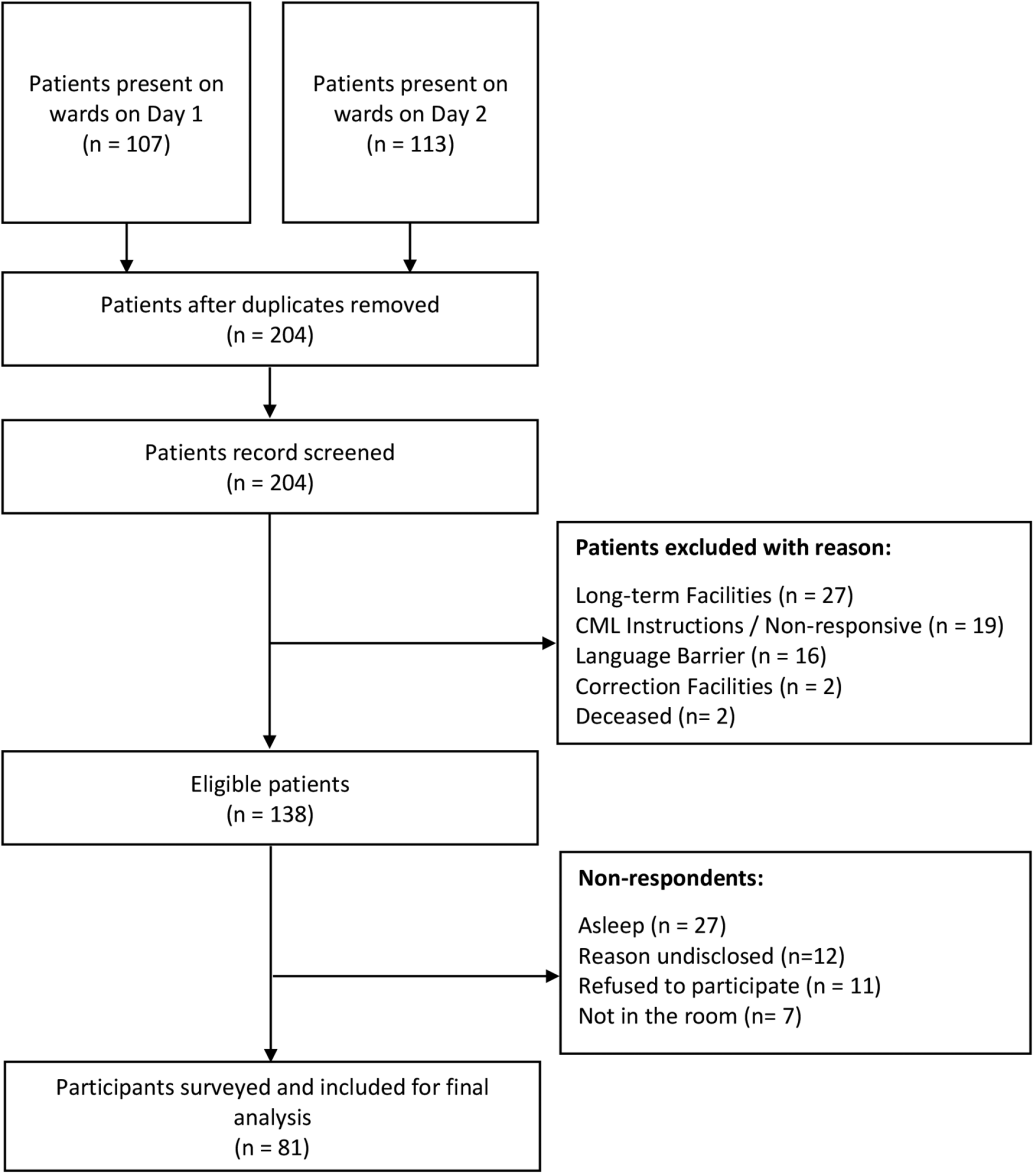
Recruitment of participants at Vancouver General Hospital internal medicine wards.

The total number of IM CTU patients present on day 1 and day 2 of the study were 107 and 113 respectively. Sixteen patients who were surveyed on day 1 remained on the ward on day 2 and were therefore excluded from the survey to avoid duplicate responses. Other exclusions included patients from LTCF (*n* = 27), patients who were non-responsive and/or study staff were given instruction not to approach (*n* = 19), patients with a language barrier for whom no interpreter was present (*n* = 16), patients from a corrections facility (*n* = 2), and patients who were deceased (*n* = 2). Most common reasons for non-response of eligible patients were “patient asleep” at the time of surveying (*n* = 27) and reasons not captured (*n* = 12). Research staff attempted contact with patients with a language barrier and/or asleep three times. Patients not surveyed due to “Language Barriers” are defined as those who could not complete the survey in English or French, and no interpreter was present to act as translator. No patients requested to complete the survey in French. Therefore, 81 of the 138 eligible patients were surveyed. Figure 1 (Recruitment of participants at Vancouver General Hospital Internal Medicine Wards) outlines the patient flowchart from identification to the final cohort of participants.

### Participant characteristics

Tables 1 provides characteristics of study participants and eligible patients. The median (IQR) age of respondents was 70 (57–83) years old, with a median age of 69 for males and 74 for females. The skewness of the normality test on age was −0.4 indicating that the distribution is skewed to the left. The kurtosis of the test was found to be −0.5, indicating a playkurtic distribution.

**Table 1:** Characteristics of survey participants.

Characteristics	Total (*n* = 81)	Male (*n* = 45, 55.6%)	Female (*n* = 36, 44.4%)
**Age (years)**			
Median	70	69	74
Range	30–98	30–94	35–98
IQR	57–83	56–78	57–86
**RRAS, *n* (%)**			
High	22 (27.2%)	10 (12.3%)	12 (14.8%)
Moderate	36 (44.4%)	19 (23.5%)	17 (21.0%)
Low	17 (21.0%)	11 (13.6%)	6 (7.4%)

RRAS, readmission risk assessment score; 6 (7.4%) with unspecified RRAS.

The gender proportion amongst eligible participants was 51% female to 49% male. The final surveyed sample was 44% female and 56% male. Finally, 71% of participants scored “moderate” to “high” RRAS (44% moderate RRAS, 27% high RRAS), and the proportion was equal between males and females.

### Mobile phone accessibility

Among study participants, 85.2% had access to a cell phone; 5% of whom shared it with a spouse or child, and 22.2% of whom had access through a proxy (an authorized third-party such as a spouse or child). Only 14.8% of participants had no phone access at all, whether shared, personal or *via* a proxy.

All participants with a mobile phone had a cellular plan; 92% (47/51) had a plan with texting and calling, and 8% had a plan with the ability to call only. Of those with a mobile phone, 25% did not have a smartphone required for app-based mHealth interventions. Tables 2A,B outline the usage and accessibility of patients.

**Table 2A:** Mobile phone access and use patterns among survey participants from general medical wards at the Vancouver General Hospital in January 2020: phone access.

Characteristics	Participants *n* (%)
Cellphone Access^a^	69 (85.2)
Personal cellphone	46 (56.8)
Shared cellphone*	4 (4.9)
Access through proxy**	18 (22.2)
No cellphone access	12 (14.8)
Total	81

^a^ Missing data: 1 didn't specify if shared or personal.

* Shared with spouse.

** Proxy: authorized individual.

**Table 2B:** Mobile phone access and use patterns among survey participants from general medical wards at the Vancouver General Hospital in January 2020: phone and plan type.

Of 51 respondents with personal/shared cellphone	*n* (%)
Type of phone^a^	
Basic phone (text/call)	8 (15.7)
Feature phone (text/call/internet)	3 (5.9)
Smartphone	38 (74.5)
IOS	21 (41.1)
Android	16 (31.4)
Other	1 (2.0)
Mobile phone plan	
Text and call	47 (92.2)
Call	4 (7.8)
No	0 (0.0)
Internet access^b^	
Data and Wi-Fi	26 (50.9)
Wi-Fi only	11 (21.6)
None	12 (23.5)

^a^ Missing data: 2 no answer.

^b^ Missing data: 2 no answer.

We also captured cellphone access and preferences in relation to patients' RRAS. In this population, patients with an RRAS of “moderate” or “high” receive regular follow-up phone calls from the hospital transition team for a period of 30 days after discharge. Participants who had a “High” RRAS all had cell phone access, with a majority owning personal cell phones. The “Low” RRAS group had a similar distribution. The “Moderate” risk of readmission group had the highest percentage of non-phone owners (Table 3).

**Table 3:** Distribution of phone ownership/access by readmission risk assessment score.

RRAS demographic	High *n* = 22	Moderate *n* = 36	Low *n* = 17	Unspecified *n* = 8
Phone owner	16 (72.7%)	21 (58.3%)	11 (64.7%)	3 (37.5%)
Access *via* proxy	6 (27.3%)	7 (19.4%)	5 (29.4%)	1 (12.5%)
No phone access	0 (0.0%)	8 (22.2%)	1 (5.9%)	2 (25.0%)

RRAS, readmission risk assessment score.

### Cellphone ownership patterns

Table 4 summarizes the mobile phone ownership patterns among participants. The median age of phone owners (66 years) is notably lower than the median age of non-phone owners (76 years) or proxy-dependent phone owners (84 years). Of the 81 survey respondents, 71.1% of males (32/45) and 52.8% of females (19/36) owned a mobile phone. More females (19.4%) than males (11.1%) did not have access to a phone at all, either personally or by proxy. Around 73% of the participants use their phone to text, with the majority of texters being females. Approximately a quarter (22.2%) of participants who owned phones had previous experience texting a healthcare provider, most of which refers to one-way communication—meaning patients are only receiving texts in the form of reminders or information. The percentage of phone owners who previously texted a healthcare provider is lower amongst male respondents (19.0%) in comparison to females (32.2%); however, given the opportunity, both genders expressed that they would use mHealth services in the future, irrespective of age or accessibility.

**Table 4:** Distribution of gender and age of survey participants by phone ownership/access.

Responses	Overall median (IQR)	Male (*N_t_* = 45)	Female (*N_t_* = 36)
Phone owners	51 (63.0%)	32 (71.1%)	19 (52.8%)
Age (years)	66 (56–72)		
Access *via* proxy	18 (22.2%)	8 (17.8%)	10 (27.8%)
Age (years)	84 (71–91)		
No phone access	12 (14.8%)	5 (11.1%)	7 (19.4%)
Age (years)	76 (64–85)		
Communicate *via* text	37 (72.5%)	22 (68.8%)	15 (79.0%)
Communicate with HCP *via* text	18 (22.2%)	8 (19.0%)	10 (32.2%)

*N_t_*, total number; HCP, healthcare providers; SD, standard deviation. This table present the breakdown of phone ownership type, distribution of texters, and patient who texted their HCP by gender.

**Figure 2:**
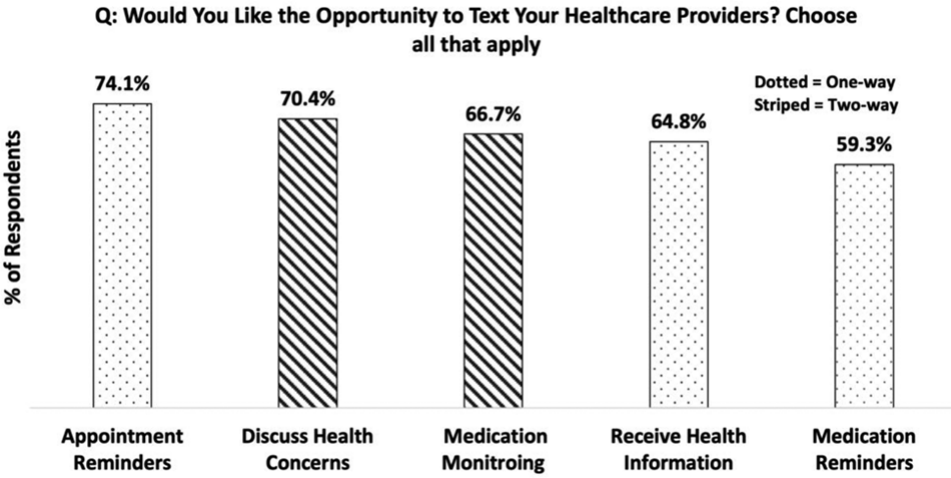
Participants preference for one-way versus two-way communication with HCP.

The level of interest of those seeking the opportunity to text their HCP was high, with 72% (53/74) of participants said they would and 28% (or 21/74) said they would not. As for the specific reasons for communicating through text with their HCP, the responses were divided into “one-way” and “two-way” communication (Figure 2). One-way indicating that patients are only receiving text, and two-way meaning that patients can both send SMS to and receive SMS from their healthcare providers. Except for receiving appointment reminders (one-way), participants preferred two-way communication, which includes capacity for medication monitoring (such as reporting side effects and requesting prescription refills) (67.9%), and to discuss healthcare concerns (71.1%). Participants indicated that they would like the opportunity to receive one-way texts in the form of medication (60.4%) and appointment reminders (75.5%) and receiving standard health information relating to their condition (66.0%).

**Figure 3:**
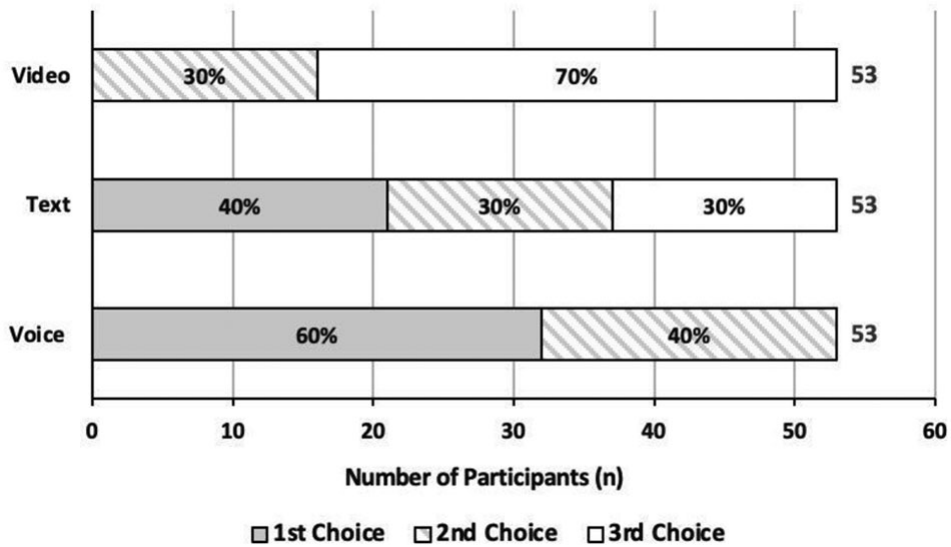
Participants' relative preferred modalities of communication using their mobile phone.

Figure 3 outlines the most frequently used communication methods. Voice call was the most preferred communication method by participants, followed by SMS/texting; with video call being the least preferred. Patients appear bimodal on video preferences, either second choice (30%) or last choice (70%) but not first. Preference for text messaging was most varied.

### Analysis of phone ownership and RRAS

Participants with high and moderate RRAS scores were significantly more likely to be primary phone owners (*P* = 0.036 and *P* = 0.007, respectively) compared to participants with low RRAS scores (*P* = 0.383). Additionally, participants with high and moderate RRAS scores were more significantly likely to have access to phones *via* a proxy (*P* = 0.053 and *P* = 0.006, respectively) compared to participants with low RRAS scores (*P* = 0.254). A Chi-Square test examined the relation between high RRAS scores and owning phones. The relation between these variables was slightly significant, *X*^2^ (2, *N* = 16) = 4.813, *P* = 0.090. Furthermore, the same test was conducted for participants with moderate RRAS scores. The relationship between moderate RRAS scores and owning a phone was significant, *X*^2^ (2, *N* = 22) = 8.514, *P* = 0.014. Similar to findings from the linear regression model, participants with low RRAS score were less likely to own phones, *X*^2^ (2, *N* = 11) = 1.358, *P* = 0.507.

## Discussion

The present study provides the first report of mobile phone ownership, accessibility, and preferences among adult general medicine inpatients of a large urban hospital within western Canada. The study results reveal that mobile phone ownership and access is generally high among this population, with slightly lower ownership among older patient groups and women. These results are consistent patients and among females, but often offset by sharing phone with a number of reports suggesting that mobile phone ownership is increasingly high and slightly varying across demographic groups. However, many of these studies were published before 2015, a considerable time ago given the past decade boom in cell phone ownership growth, and only one study reports on the state of patient phone ownership in Canada (eastern Canada, outpatient mental health) (21–26). Furthermore, the majority (92%) of mobile phone owners were observed to have cellular plans with the ability to call and to text, and although most participants reported using their mobile phones regularly, few indicated texting an HCP in the past. We found cell phone-owners and non-cell phone owners alike would like the opportunity to use mobile phones to communicate with an HCP during their care process, suggesting a welcoming desire for the integration of technology-enabled patient-to-provider communication streams into care practices, as is being observed worldwide (27–29).

### Device ownership

A small majority of survey respondents (56%) reported owning their own cell phones. This is significantly lower than government reports of phone ownership in Canada and reveals a gap in the penetration of such devices into the population who stays in the hospital (2, 30). This discrepancy may be the result of the participants being mostly elderly, or other influences such as sociodemographic factors that were not measured here but have been reported to correlate with ownership (31). Fortunately, respondents who did not own a phone were very likely to have access to one (shared or proxy), somewhat mitigating the concern that broadly deployed cell phone based medical interventions would be inaccessible to nearly half of patients who require general hospital-based care. Of the owned phones, most of these devices utilize the iOS or Android operating systems (74.5%). These results are consistent with broad market trends in Canada and beyond, where these two operating systems dominate after taking over from basic phones that initially expanded the mobile market (32). All things considered, an important proportion of medical populations (14.8%) could not access a phone at all and therefore are not able to participate in increasingly popular mobile health interventions.

### Mode of service delivery

In recent years, many medical technology developers have focused on smartphone apps as the mode of service delivery (33–35). However, our data show that not all medical patients have access to the internet on their mobile devices, nor may know how to use “apps”. In fact, 25% (12/51) of participants could not access the internet through their phone, either through Wi-Fi or Data, limiting patient access to app/internet-dependent mHealth services. Moreover, given the predominant participant preference for voice calls, it is plausible to presume that the uptake of complicated phone apps would be significantly low, particularly in the elderly population. The largest proportion of participants in the current study can access a basic mobile device that can send and receive phone calls and text messages, suggesting that these two modes of mobile communication should be pursued to ensure digital tools meet the abilities of the Canadian population. When asked about their text-based communication preferences, participants expressed a higher inclination for two-way communication, with one participant stating that “one-way doesn't make sense. It must be two-way”. Indeed, in an external meta-analysis, 2-way texting interventions were found to be more effective than 1-way interventions at improving medication treatment adherence (36). Multiple other patients indicated that they would prefer to text their HCP regarding medication concerns, scheduling, and more (i.e., “two-way”; including discussing medication side-effects, need for a refill, etc.), in addition to receiving medication or appointment reminder messages (“one-way”). Therefore, although two-way communication was preferred, as reflected in our results, participants still would like the opportunity to receive one-way texts. This suggests that a mixed approach to text based mobile health, is most appropriate and desired.

### Barriers to accessibility: Gender and age

Our findings highlight known inequities in access to digital healthcare (37). We found that more male participants owned and had access to mobile phones than females and that younger individuals were more likely to own phones. These findings are in line with other studies that report women to be disadvantaged in terms of mobile internet use and less likely to own a mobile phone (38, 39). They are also in line with Gordon and Hornbrook, among others, who point to a digital divide between older and younger populations regarding device ownership and health information preferences (40–42). Contrarily, in a study conducted in Kenya among HIV participants, women had equal or more participation than their male counterparts when using a text messaging based mHealth tool (43). Evidence for this female-based preference for texting is provided by our study. Of those who did own phones, females were more likely to communicate by text. Additionally, when asked about the opportunity to text their HCPs, only 17% of women declined, compared to 33% of men. The observed preference for female participants to text is consistent with additional literature, which reports that more female than male participants are “mediated communicators” who more habitually communicate using their mobile phones and have a higher preference for direct two-way communication with their health care team (44). Notably, a male respondent enthusiastically reported that mobile phone use in healthcare “is the future” and that although he is unable to text, his female partner would utilize a mHealth intervention on his behalf. This sentiment was shared by other participants. These observations point to potential demographic group and context specific preferences for the technologic basis of mHealth tools. That is, text messages may be preferred among certain demographic groups or contexts, and perhaps other modalities (applications, websites) for others. This is useful information for researchers and clinical teams to know when deploying phone based digital health tools that engage multiple subgroups.

### mHealth and transitional care

As detailed above, the survey results suggest that most participants (72%) are open to using their phones to communicate about their healthcare. For inpatient populations, using phones to communicate with care providers during the hospital stay is likely redundant and unnecessary, however, they are likely to be of value after discharge where there is a current struggle with continuity of care and hospital readmission, worldwide (45–47). mHealth initiatives could provide a transitional care communication stream to all patients discharged from the hospital regardless of their RRAS. Accordingly, our study found that participants with high and moderate RRAS scores were significantly more likely to have access to mobile phones either as primary owners or through proxy, although the reason for this is not clear. This finding provides support for successful uptake of targeted mHealth interventions that address hospital readmissions among participants who are most likely to be readmitted [i.e., with high and moderate “Risk of Readmission Scores” (RRAS)].

### Strengths

This study has an above average survey response rate (58.7% after screening for eligibility) (48). The high response rate was likely due to the distribution method, where surveys were researcher administered directly to patients on the ward. We aimed to ensure the highest inclusion of patients-in-ward as possible, including patients who may have had limitations in reading or answering the survey, understanding the survey questions, using the tablet/phone, and other potential hindrances to completing the survey on their own—without being coercive. Another strength of the current study is that the survey was audited by a consortium of health professionals, including a mHealth professional, an HCP working on the ward, and a member from the hospitals initiatives team to ensure relevancy to study objectives and the hospital's priorities for patient care. Lastly, due to the nature of the public health care system in Canada where the general population has access to health care services, the patient population within the hospital is representative of the local general population.

### Limitations

The current study has several limitations that are important for the reader to consider. First, the study utilized a cross-sectional survey approach that does not capture seasonal/time-based changes in the patient population. We selected this approach due to its appropriateness for providing the target time-point descriptive data and were not aiming to investigate time-based changes in phone ownership. While we suspect that the broad diversity of patients typically cared for in the selected setting helps to offset some seasonal/time-based changes in the patient population and thus outcome measures, they are still important to consider and may impact phone ownership (49–51). Second, the sample size is limited due to research activity cessation in response to the COVID-19 pandemic. That said, given the surge in virtual health technology and telemedicine implementation and dependence spurred by the pandemic, the results of this study provide a unique and very relevant baseline measurement of phone ownership, accessibility, and preferences. Third, patients with language barriers to research staff were not considered for participation as it prevented them from understanding survey questions and/or providing informed consent. In future iterations of this study, translation and interpretation services could be deployed to better understand the access and phone preferences of this population. Fourth, although initial demographic comparisons show no differences between participant responders and non-responders in terms of gender and age, with a 59% response rate, we expect some nonresponse bias (see Table 1). Finally, we surveyed patients in the IM CTUs where the results may not be generalizable to other urban hospital inpatient groups, such as surgical patients. This study population was conveniently sampled which may have introduced biases through patient selection. This specific population was selected as they are part of an active and already funded project by the UBC mHealth Research Group. Future iterations of this survey should include a variety of inpatient hospital wards in an urban clinical setting.

### Next steps

It is worthwhile for future research to explore the source of phone ownership (for instance, a direct purchase or a hand-me-down device) as it may have implications to understanding the level of digital literacy among smartphone owners and efficacious use of digital health interventions. Additionally, an investigation of the disease profile of similar study populations would help to determine if there is a relationship between disease types and phone ownership/accessibility and ultimately understand the feasibility of phone based medical interventions for patients based upon diagnosis. Finally, it would be intriguing to investigate the impact of the COVID-19 pandemic on phone ownership, accessibility, and preferences in this target population, and generally, using a post-pandemic survey. Our team suspects that the pressure placed on global populations by public health initiatives encouraged the adoption of digital devices and may have increased phone ownership and accessibility, and opened more of the population to the idea of using digital tools to receive healthcare.

## Conclusion

mHealth solutions are likely to be useful medical innovations in the delivery of care, but concerns hindering adoption involve mobile phone ownership and disparities in cellular and internet access among patient populations. This study outlines an assessment of patient's mobile phone access and usage preferences where cellular service access among Canadian acute medical ward patients was high yet diverse. Insight gained through understanding of mobile phone use patterns of patient populations may support health service planners to develop interventions that are sustainable, current, and patient-centered. Such considerations are even more critical during the current global pandemic as vulnerable and marginalized communities face disparities in access to care and unique challenges in accessing community resources. We hope findings from this study can be employed to inform interventions aimed at supporting vulnerable inpatients populations and hospital discharge protocols in Canada. Although this paper presents novel data that is region-specific, results can potentially be translated to similar contexts globally. The contribution of this paper is in uncovering positive values on mobile phone penetration and cellular service access. Furthermore, it offers a detailed breakdown of gender and age disparities in access and mobile phone usage patterns. This has implications for the use of mobile phones for the provision of healthcare and development.

## Data Availability

In compliance with the university ethics Research Ethics Board, the aggregated survey data supporting the conclusions of this article will be made available by the authors, without undue reservation. Requests to access the remaining data can be directed to the corresponding author.

## References

[1] Fourth Industrial Revolution. World economic forum. Available at: https://www.weforum.org/focus/fourth-industrial-revolution (Accessed April 02, 2021).

[2] Smartphone ownership is growing rapidly around the world, but not always equally. Pew research center. Available at: https://www.pewresearch.org/global/2019/02/05/smartphone-ownership-is-growing-rapidly-around-the-world-but-not-always-equally/ (Accessed April 02, 2021).

[3] Market Research Reports. Digital health market revenue of US$536.6 bn by the end of 2025: technology advancement and innovation - press release - digital journal. Market research reports (2017). Available at: http://www.digitaljournal.com/pr/3518555 (Accessed April 02, 2021).

[4] Ryu S. mHealth: new horizons for health through mobile technologies: based on the findings of the second global survey on ehealth (global observatory for ehealth series, volume 3). Healthc Inform Res. (2012) 18(3):231. 10.4258/hir.2012.18.3.231

[5] Salud OM de la. WHO | medical device – full definition. World Health Organization (2013). p. 1. Available at: https://www.who.int/medical_devices/full_deffinition/en/ (Accessed April 02, 2021).

[6] About the digital health center of excellence | FDA. Available at: https://www.fda.gov/medical-devices/digital-health-center-excellence/about-digital-health-center-excellence (Accessed May 10, 2021).

[7] Starfield B Shi L Macinko J. Contribution of primary care to health systems and health. Milbank Q. (2005) 83:457–502. 10.1111/j.1468-0009.2005.00409.x 16202000 PMC2690145

[8] Lester RT Ritvo P Mills EJ Kariri A Karanja S Chung MH Effects of a mobile phone short message service on antiretroviral treatment adherence in Kenya (WelTel Kenya1): a randomised trial. Lancet. (2010) 376(9755):1838–45. 10.1016/S0140-6736(10)61997-6 21071074

[9] Larbi D Randine P Årsand E Antypas K Bradway M Gabarron E. Methods and evaluation criteria for apps and digital interventions for diabetes self-management: systematic review. J Med Internet Res. (2020) 22(7):1–13. 10.2196/18480 PMC7381260 32628125

[10] Wang Y Xue H Huang Y Huang L Zhang D. A systematic review of application and effectiveness of mhealth interventions for obesity. Adv Nutr. (2017) 8(3):449–62. 10.3945/an.116.014100 28507010 PMC5421120

[11] Hu J Gonsahn MD Nerenz DR. Socioeconomic status and readmissions: evidence from an urban teaching hospital. Health Aff. (2014) 34(5):778–85. 10.1377/hlthaff.2013.0816 24799574

[12] Arora S Peters AL Burner E Lam CN Menchine M. Trial to examine text message-based mhealth in emergency department patients with diabetes (TExT-MED): a randomized controlled trial. Ann Emerg Med. (2014) 63(6):745–754.e6. 10.1016/j.annemergmed.2013.10.012 24225332

[13] Nurgalieva L O’Callaghan D Doherty G. Security and privacy of mhealth applications: a scoping review. IEEE Access. (2020) 8:104247–68. 10.1109/ACCESS.2020.2999934

[14] Gurupur VP Wan TTH. Challenges in implementing mhealth interventions: a technical perspective. mHealth. (2017) 3:32. 10.21037/mhealth.2017.07.05 28894742 PMC5583043

[15] Huygens MWJ Vermeulen J Swinkels ICS Friele RD Van Schayck OCP De Witte LP. Expectations and needs of patients with a chronic disease toward self-management and ehealth for self-management purposes. BMC Health Serv Res. (2016) 16(1):232. 10.1186/s12913-016-1484-5 27391471 PMC4938915

[16] Jennings L Lee N Shore D Strohminger N Allison B Conserve DF U.S. minority homeless youth's access to and use of mobile phones: implications for mhealth intervention design. J Health Commun. (2016) 21(7):725–33. 10.1080/10810730.2015.1103331 27232544

[17] Stevenson JK Campbell ZC Webster AC Chow CK Tong A Craig JC Ehealth interventions for people with chronic kidney disease. Cochrane Database Syst Rev. (2019) 2019(8):2–5. 10.1002/14651858.CD012379 PMC6699665 31425608

[18] Choi WS Choi JH Oh J Shin IS Yang JS. Effects of remote monitoring of blood pressure in management of urban hypertensive patients: a systematic review and meta-analysis. Telemed e-Health. (2020) 26(6):744–59. 10.1089/tmj.2019.0028 31532328

[19] CIHI. All Patients Readmitted to Hospital. Available at: https://yourhealthsystem.cihi.ca/hsp/inbrief.#!/indicators/006/all-patients-readmitted-to-hospital/;mapC1;mapLevel2;provinceC5001;trend(C1,C5001);/ (Accessed April 04, 2021).

[20] Brooten D Brown LP Munro BH York R Cohen SM Roncoli M Early discharge and specialist transitional care. Image J Nurs Scholarsh. (1988) 20(2):64–8. 10.1111/j.1547-5069.1988.tb00032.x 3378819

[21] Firth J Cotter J Torous J Bucci S Firth JA Yung AR. Mobile phone ownership and endorsement of “mhealth” among people with psychosis: a meta-analysis of cross-sectional studies. Schizophr Bull. (2015) 42(2):448–55. 10.1093/schbul/sbv132 26400871 PMC4753601

[22] Langford AT Solid CA Scott E Lad M Maayan E Williams SK Mobile phone ownership, health apps, and tablet use in US adults with a self-reported history of hypertension: cross-sectional study. JMIR Mhealth Uhealth. (2019) 7(1):1–2. 10.2196/12228 PMC6682274 31344667

[23] Zurovac D Otieno G Kigen S Mbithi AM Muturi A Snow RW Ownership and use of mobile phones among health workers, caregivers of sick children and adult patients in Kenya: cross-sectional national survey. Global Health. (2013) 9(1):20. 10.1186/1744-8603-9-20 23672301 PMC3695884

[24] Torous J Chan SR Yee-Marie Tan S Behrens J Mathew I Conrad EJ Patient smartphone ownership and interest in mobile apps to monitor symptoms of mental health conditions: a survey in four geographically distinct psychiatric clinics. JMIR Ment Health. (2014) 1(1):1–2, 6. 10.2196/mental.4004 PMC4607390 26543905

[25] Torous J Friedman R Keshavan M. Smartphone ownership and interest in mobile applications to monitor symptoms of mental health conditions. JMIR Mhealth Uhealth. (2014) 2(1):2, 6–7. 10.2196/mhealth.2994 PMC4114412 25098314

[26] Di Matteo D Fine A Fotinos K Rose J Katzman M. Patient willingness to consent to mobile phone data collection for mental health apps: structured questionnaire. JMIR Ment Health. (2018) 5(3):2–3, 7. 10.2196/mental.9539 PMC6135964 30158102

[27] Marcolino MS Oliveira JA D'Agostino M Ribeiro AL Alkmim MB Novillo-Ortiz D. The impact of mhealth interventions: systematic review of systematic reviews. JMIR Mhealth Uhealth. (2018) 6(1):7–8. 10.2196/mhealth.8873 PMC5792697 29343463

[28] de la Harpe R McLean N. Integration of mhealth information and communication technologies into the clinical settings of hospitals in sub-saharan Africa: qualitative study (preprint). JMIR Mhealth Uhealth. (2020) 9(10):6–9. 10.2196/26358 PMC8552092 34643540

[29] Mbuthia F Reid M Fichardt A. mHealth communication to strengthen postnatal care in rural areas: a systematic review. BMC Pregnancy Childbirth. (2019) 19(1):6–8. 10.1186/s12884-019-2531-0 31694578 PMC6836428

[30] Statistics Canada. Smartphone use and smartphone habits by gender and age group. Statistics Canada. Government of Canada (2021). Available at: https://www150.statcan.gc.ca/t1/tbl1/en/tv.action?pid=2210011501 (Accessed July 19, 2022).

[31] Oshima SM Tait SD Thomas SM Fayanju OM Ingraham K Barrett NJ Association of smartphone ownership and internet use with markers of health literacy and access: cross-sectional survey study of perspectives from project place (population level approaches to cancer elimination). J Med Internet Res. (2021) 23(6):1–16. 10.2196/24947 PMC8262672 34106076

[32] Published by Statista Research Department, 23 M. smartphone sales by year by OS 2009-2018. Statista (2022). Available at: https://www.statista.com/statistics/266219/global-smartphone-sales-since-1st-quarter-2009-by-operating-system/ (Accessed July 19, 2022).

[33] Kao C-K Liebovitz DM. Consumer mobile health apps: current state, barriers, and future directions. PM R. (2017) 9(5):S106–S115. 10.1016/j.pmrj.2017.02.018 28527495

[34] Boudreaux ED Waring ME Hayes RB Sadasivam RS Mullen S Pagoto S. Evaluating and selecting mobile health apps: strategies for healthcare providers and healthcare organizations. Transl Behav Med. (2014) 4(4):363–71. 10.1007/s13142-014-0293-9 25584085 PMC4286553

[35] mHealth apps market size & share report, 2022-2030. Available at: https://www.grandviewresearch.com/industry-analysis/mhealth-app-market&hash;:∼:text=The&per;20global&per;20mHealth&per;20apps&per;20market&per;20is&per;20expected&per;20to&per;20grow&per;20at,share&per;20of&per;2096.8&per;25&per;20in&per;202021 (Accessed July 19, 2022).

[36] Wald DS Butt S Bestwick JP. One-way versus two-way text messaging on improving medication adherence: meta-analysis of randomized trials. Am J Med. (2015) 128(10):1139.e1–.e5. 10.1016/j.amjmed.2015.05.035 26087045

[37] Women & mobile: a global opportunity a study on the mobile phone gender gap in low and middle-income countries. Available at: http://www.mobileasiacongress.com (Accessed April 03, 2021).

[38] Khatun F Heywood AE Hanifi SMA Rahman MS Ray PK Liaw ST Gender differentials in readiness and use of mhealth services in a rural area of Bangladesh. BMC Health Serv Res. (2017) 17(1):573. 10.1186/s12913-017-2523-6 28821243 PMC5563057

[39] Striving and surviving: exploring the lives of women at the base of the pyramid. Available at: http://www.mwomen.org (Accessed May 02, 2021).

[40] Gordon NP Hornbrook MC. Older adults’ readiness to engage with ehealth patient education and self-care resources: a cross-sectional survey. BMC Health Serv Res. (2018) 18(1):220. 10.1186/s12913-018-2986-0 29587721 PMC5872546

[41] Kumar D Hemmige V Kallen M Giordano T Arya M. Mobile phones may not bridge the digital divide: a look at mobile phone literacy in an underserved patient population. Cureus. (2019) 11(2). 10.7759/cureus.4104 PMC6476614 31057998

[42] Mubarak F Suomi R. Elderly forgotten? Digital exclusion in the information age and the rising grey digital divide. Inquiry. (2022):59:469580221096272. 10.1177/00469580221096272 35471138 PMC9052810

[43] van der Kop ML Karanja S Thabane L Marra C Chung MH Gelmon L In-depth analysis of patient-clinician cell phone communication during the WelTel Kenya1 antiretroviral adherence trial. PLoS One. (2012) 7(9):1–8. 10.1371/journal.pone.0046033 PMC3457960 23049928

[44] Kimbrough AM Guadagno RE Muscanell NL Dill J. Gender differences in mediated communication: women connect more than do men. Comput Human Behav. (2013) 29(3):896–900. 10.1016/j.chb.2012.12.005

[45] Jencks SF Williams MV Coleman EA. Rehospitalizations among patients in the medicare fee-for-service program. N Engl J Med. (2009) 361(3):311. 10.1056/NEJMc090911 19339721

[46] Schuster C Hurlburt A Tam P Staples JA. Unplanned hospital readmissions in British Columbia. B C Med J. (2018) 60(5):263–7. 10.14288/1.0412183

[47] Nicaise P Giacco D Soltmann B Pfennig A Miglietta E Lasalvia A Healthcare system performance in continuity of care for patients with severe mental illness: a comparison of five European countries. Health Policy. (2020) 124(1):25–36. 10.1016/j.healthpol.2019.11.004 31831211

[48] Baruch Y Holtom BC. Survey response rate levels and trends in organizational research. Hum Relations. (2008) 61(8):1139–60. 10.1177/0018726708094863

[49] Canadian Medical Association. General internal medicine profile. Ottawa: Canadian Medical Association (2019).

[50] Argha A Savkin A Liaw S-T Celler BG. Effect of seasonal variation on clinical outcome in patients with chronic conditions: analysis of the commonwealth scientific and industrial research organization (CSIRO) national telehealth trial. JMIR Med Inform. (2018) 6(1):3–9. 10.2196/medinform.9680 PMC5878365 29549068

[51] Stewart S Keates AK Redfern A McMurray JJ. Seasonal variations in cardiovascular disease. Nat Rev Cardiol. (2017) 14(11):654–64. 10.1038/nrcardio.2017.76 28518176

[52] Staples JA Wiksyk B Liu G Desai S van Walraven C Sutherland JM. External validation of the modified LACE+, LACE+, and LACE scores to predict readmission or death after hospital discharge. J Eval Clin Pract. (2021) 27(6):1390–7. 10.1111/jep.13579 33963605

[53] Smartphone penetration in Canada as share of population 2018-2025. Statista. Available at: https://www.statista.com/statistics/472054/smartphone-user-penetration-in-canada/ (Accessed April 25, 2022).

[54] RStudio Team. RStudio: integrated development for R. RStudio, PBC, Boston, MA (2020). Available at: http://www.rstudio.com/.

